# Parental Attitudes Toward Childhood and Adolescent Vaccination in Poland in 2026: Vaccination Uptake and Retrospectively Reported Changes Since the Pre-COVID-19 Period

**DOI:** 10.3390/vaccines14100906

**Published:** 2026-10-17

**Authors:** Magdalena Konieczny, Izabela Gąska, Elżbieta Kaczmar, Dorota Bądziul, Andrzej M. Fal, Dorota Kiedik, Łukasz Rypicz

**Affiliations:** 1 Medical Institute, Jan Grodek State University in Sanok, 38-500 Sanok, Poland; magdalenakonieczny@wp.pl (M.K.); igaska@up-sanok.edu.pl (I.G.); ekaczmar@up-sanok.edu.pl (E.K.); 2 Polish Society of Public Health, 51-618 Wroclaw, Poland; amfal@wp.pl (A.M.F.); dorota.kiedik@umw.edu.pl (D.K.); 3 Department of Medical Biology, Institute of Medical Sciences, Medical College of Rzeszow University, Rejtana 16 C, 35-959 Rzeszow, Poland; dbadziul@ur.edu.pl; 4 Faculty of Medicine, Collegium Medicum, Cardinal Stefan Wyszyński University in Warsaw, 01-938 Warsaw, Poland; 5 Department of Public Health, Division of Public Health, Faculty of Health Sciences, Wroclaw Medical University, 50-367 Wroclaw, Poland

**Keywords:** vaccination, childhood vaccination, parental attitudes

## Abstract

**Background/Objectives:** Parental attitudes toward vaccination influence the timely and complete immunization of children, including uptake of recommended vaccines. This study assessed self-reported compliance with mandatory vaccination, use of recommended vaccines, and parental attitudes toward childhood and adolescent vaccination in Poland, including regional variation and retrospectively reported changes since the pre-COVID-19 period. **Methods:** A nationwide cross-sectional CAWI survey was conducted in the first quarter of 2026 among 1568 parents of children aged 0–18 years. Analyses included mandatory and recommended vaccination uptake, reasons for vaccinating or not vaccinating, attitudes toward vaccination, and differences across six Polish macroregions. **Results:** Overall, 95.0% of respondents reported vaccinating their child according to the mandatory schedule. Recommended vaccines were used regularly by 35.4%, selectively by 36.8%, and not used by 27.8%. Among parents reporting non-compliance with mandatory vaccination (N = 79), the most common reasons were concerns about adverse effects (70.9%), vaccine safety (50.6%), distrust of pharmaceutical companies (41.8%), and long-term effects (39.2%). No significant regional differences were found in current uptake of mandatory, recommended, or combination vaccines. However, current vaccine acceptance was retrospectively rated lower than before the COVID-19 pandemic in all regions, and the magnitude of the retrospectively reported difference varied significantly between regions (*p* = 0.013). **Conclusions:** High reported uptake of mandatory vaccination coexists with selective use of recommended vaccines and persistent safety concerns. Transparent, evidence-based communication addressing parental concerns may help sustain vaccine confidence and support informed use of recommended vaccines.

## 1. Introduction

Vaccination is one of the most effective public health interventions, but its benefits depend on sustained public acceptance and timely uptake [1]. In childhood vaccination, decisions are largely made by parents or legal guardians. Vaccine hesitancy (VH) encompasses a spectrum ranging from full acceptance to selective uptake, delay, or refusal and may vary according to time, place, and vaccine type [2]. Vaccination decisions are influenced by confidence in vaccines and healthcare systems, perceived disease risk, barriers to access, information seeking, and collective responsibility [3]. Parental VH is particularly relevant because it may contribute to delayed or missed childhood vaccination [4]. A meta-analysis estimated substantial parental hesitancy toward routine childhood vaccination worldwide [5], while European research demonstrated marked between-country differences in vaccine confidence, with Poland identified as a country with a relatively lower level of confidence [6].

Evidence from Poland similarly indicates that parental vaccination attitudes are heterogeneous. Although confidence in mandatory vaccination has generally remained high, concerns regarding vaccine safety and effectiveness persist among some parents [7]. Previous Polish studies have linked vaccination attitudes to sources of information, previous experiences with adverse events following immunization, perceptions of medical qualification, sociodemographic characteristics, and individual experiences [8,9,10,11]. Concerns about adverse events and vaccine effectiveness have also been identified among the most frequently reported barriers to vaccination [12].

The COVID-19 pandemic created an additional context for vaccination decision-making, characterized by intensive public debate, rapidly evolving vaccine information, and widespread exposure to both information and misinformation. International evidence does not indicate a uniform effect of the pandemic on attitudes toward routine childhood vaccination [13,14]. In Poland, post-pandemic qualitative research has identified concerns about adverse events, confidence in elements of the vaccination program, and individual assessments of benefits and risks as relevant factors shaping parental views [15]. These findings support the need for an updated nationwide assessment of parental attitudes toward childhood and adolescent vaccination in Poland, taking into account potential regional variation.

Against this background, the organization of childhood and adolescent vaccination in Poland provides important context for interpreting both vaccination uptake and parental attitudes. In 2025, vaccination was implemented in accordance with the National Immunization Program (Program Szczepień Ochronnych, PSO), announced annually by the Chief Sanitary Inspector. The program included both mandatory and recommended vaccinations for children and adolescents. Mandatory vaccinations were financed from public funds and provided free of charge to individuals covered by the program until the age of 19 years. They included vaccination against tuberculosis, hepatitis B, rotavirus infection, diphtheria, tetanus, pertussis, poliomyelitis, Haemophilus influenzae type b, pneumococcal disease, measles, mumps, and rubella. During adolescence, the schedule also included booster doses against diphtheria, tetanus, and pertussis, followed by a booster dose against diphtheria and tetanus [16].

Despite the universal and publicly funded nature of mandatory childhood vaccination, complete adherence to the vaccination schedule has not been achieved in all age groups. According to the most recent report of the National Institute of Public Health NIH—National Research Institute on vaccination coverage in Poland in 2024, full completion of age-required vaccinations by the third year of life was achieved in 84.4% of children (253,845 individuals). Among mandatory primary vaccinations, the highest coverage was reported for hepatitis B vaccination (94.3%), whereas lower levels were observed for some multidose vaccination schedules, including vaccination against Haemophilus influenzae type b [17]. These data indicate that, even within the mandatory vaccination system, differences in the completeness of vaccination schedules remain evident.

In parallel with mandatory vaccination, the Polish immunization program includes a range of recommended vaccines. These included, among others, vaccines against human papillomavirus (HPV), influenza, meningococcal disease, varicella, hepatitis A, tick-borne encephalitis, and COVID-19. Some recommended vaccinations were publicly funded, including HPV and COVID-19 vaccination, as well as influenza vaccination in specified population groups, whereas the remaining vaccines generally required out-of-pocket payment, except for individuals meeting defined reimbursement criteria [18].

The distinction between mandatory and recommended vaccination is particularly relevant when assessing parental vaccination behavior, as uptake of recommended vaccines may depend to a greater extent on individual risk perception, confidence in vaccine safety and effectiveness, access, and willingness to incur potential costs. At the same time, the absence of a unified monitoring system covering all recommended vaccinations limits direct comparisons of their uptake. Available data indicate an increase in HPV vaccination uptake following the introduction of the publicly funded vaccination program, whereas influenza vaccination remained among the least frequently administered recommended vaccines. In the 2024/2025 season, influenza vaccination coverage was approximately 4.3% among children aged 0–4 years and 3.6% among those aged 5–14 years [19].

COVID-19 vaccination represented an additional component of the recommended vaccination framework. In 2025, it remained voluntary and was available free of charge to children from 6 months of age and to adolescents. Current recommendations particularly emphasized its importance for individuals with risk factors for severe COVID-19, although vaccination was also available to healthy individuals [20]. Taken together, the coexistence of a publicly funded mandatory vaccination schedule and a more heterogeneous system of recommended vaccinations provides a relevant framework for assessing whether high adherence to mandatory vaccination may coexist with selective uptake of recommended vaccines and persistent concerns regarding vaccine safety.

The aim of this study was to assess self-reported compliance with mandatory vaccination, the use of recommended vaccines, and parental attitudes toward childhood and adolescent vaccination in Poland. The specific objectives were: (1) to describe the reasons for compliance and non-compliance with mandatory vaccination; (2) to assess vaccination-related attitudes and concerns; (3) to compare self-reported vaccination uptake and attitudes across six macroregions of Poland; and (4) to assess retrospectively reported changes in attitudes toward vaccination compared with the pre-COVID-19 period. The study also examined whether a high self-reported level of compliance with mandatory vaccination may coexist with selective use of recommended vaccines and persistent concerns regarding vaccine safety.

## 2. Materials and Methods

### 2.1. Study Design and Study Population

The study was conducted in Poland during the first quarter of 2026 as part of a broader research project examining parental attitudes toward vaccination. Data collection was carried out by the Pollster Research Institute. Eligible participants were adults aged 18 years or older who had at least one child aged 0–18 years. Ultimately, data from 1568 respondents were included in the analysis. The sample was constructed to reflect the distribution of selected demographic characteristics in the population of adult parents meeting the study inclusion criteria.

The study used a quantitative design and was conducted using the Computer-Assisted Web Interview (CAWI) method. The use of an online questionnaire allowed respondents to participate under convenient conditions and ensured the anonymity of their responses, which was particularly important given the sensitive nature of the study topics, including attitudes, beliefs, and opinions regarding vaccination. The questionnaire used in the study, including the original Polish version and its English translation, is provided in Supplementary File S1.

### 2.2. Sampling and Recruitment

A stratified quota sampling approach was applied. The sample structure was controlled for parental sex, including representation of both mothers and fathers, as well as age, educational level, and size of the place of residence. During data collection, the distributions of additional variables, including household financial situation and geographical location, were also monitored to reduce the risk of overrepresentation of respondents from particular areas of the country.

Participants were recruited through a professional online research panel certified under the Interviewer Quality Control Programme. For respondents with more than one child meeting the inclusion criterion (i.e., aged 0–18 years), one eligible child was randomly selected. All subsequent child-related questions referred exclusively to this randomly selected child, hereafter referred to as the reference child.

### 2.3. Research Instruments

The study used an original questionnaire developed specifically for the purposes of this research. The instrument enabled a multidimensional assessment of parental attitudes toward vaccination and the identification of factors associated with vaccination decision-making.

For the purposes of the present study, only selected data collected within the broader research project were analyzed. The analysis included respondents’ sociodemographic characteristics, information on childhood vaccination, self-reported compliance with mandatory vaccination, use of recommended vaccines, use of combination vaccines, reasons underlying vaccination-related decisions, and retrospectively reported changes in attitudes compared with the pre-COVID-19 period. Particular attention was given to regional variation in parental attitudes. Respondents were analyzed according to six macroregions of Poland. In addition, a composite measure of vaccine acceptance was developed to enable comparison between the retrospectively reported level of vaccine acceptance before the COVID-19 pandemic and that reported at the time of the survey.

### 2.4. Data Quality Control

The data collection process was subject to multistage quality control procedures. Logical consistency checks were performed to identify mutually inconsistent responses and to verify correct routing through questionnaire filter questions. Respondents classified as speeders, defined as those whose questionnaire completion time was substantially shorter than the predefined reliability threshold, were excluded from the analysis.

The data were also screened for straightlining, defined as providing identical responses across extended blocks of scale-based questions. Records showing insufficient response variability, including a standard deviation of zero within the analyzed scale-based item blocks, were reviewed and excluded in accordance with the predefined data quality criteria.

### 2.5. Statistical Analysis

The primary outcomes were: (1) self-reported compliance with the mandatory vaccination schedule (yes/no); (2) use of recommended vaccines (regularly/selectively/not at all); and (3) current self-reported attitudes toward vaccination. Secondary outcomes included the use of combination vaccines, reasons underlying vaccination-related decisions, and retrospectively reported changes in attitudes compared with the pre-COVID-19 period.

Statistical analyses were performed using STATISTICA version 13, with the level of statistical significance set at *p* < 0.05. Descriptive statistics were initially calculated, including absolute frequencies (N) and percentages (%). Associations between categorical variables were assessed using the chi-square test of independence. For ordinal variables and comparisons involving more than two independent groups, the non-parametric Kruskal–Wallis test was used, together with analysis of mean ranks.

To assess the level of vaccination acceptance, a vaccination acceptance index was calculated. The index was based on respondents’ answers regarding their attitudes toward vaccination. Individual responses were assessed using a five-point Likert scale, in which higher values indicated a higher level of vaccination acceptance. The response categories were coded as follows: 1—“No”, 2—“Rather no”, 3—“I do not know”, 4—“Rather yes”, and 5—“Yes”. The index value was calculated by aggregating responses across the analyzed questionnaire items and subsequently rescaling the result to a range of 1 to 5 points. Higher index values indicated greater vaccination acceptance.

The index constructed in this manner enabled comparison of the level of vaccination acceptance retrospectively reported by respondents for the period before the COVID-19 pandemic with that reported at the time of the study, as well as analysis of changes in attitudes toward vaccination across the individual macroregions of Poland.

Additionally, to facilitate interpretation of the results and enable comparison of the level of acceptance of individual statements concerning vaccination, including both positively and negatively worded items, the mean acceptance score for each analyzed statement was calculated on a scale ranging from 1 to 5 points. The same response coding scheme as for the vaccination acceptance index was applied, with higher values indicating a higher level of acceptance of a given statement.

### 2.6. Ethical Considerations

The study was conducted in accordance with the principles of the Declaration of Helsinki. Participants received information regarding the purpose of the study, the voluntary nature of participation, and the anonymity of their responses, and provided informed consent before beginning the survey. The study protocol was approved by the Bioethics Committee of the Jan Grodek State University in Sanok (approval No. 6/2025).

Generative AI was used solely for linguistic and stylistic editing of the manuscript. The authors reviewed and approved the final text.

## 3. Results

### 3.1. Characteristics of the Study Group

The analysis included 1568 respondents aged 19–66 years. The mean age was 42.1 years (SD = 8.4). The sex distribution was nearly equal, with women accounting for 49.9% (N = 783) of the sample and men for 50.1% (N = 785). The largest proportion of respondents lived in rural areas (41.6%; N = 652). A further 19.4% (N = 304) lived in towns with up to 50,000 inhabitants, 15.2% (N = 239) in cities with 100,000–500,000 inhabitants, while 11.9% lived in towns with 50,000–100,000 inhabitants and 11.9% in cities with more than 500,000 inhabitants.

Most respondents were in a relationship (87.4%; N = 1371), whereas 12.6% (N = 197) were single. The largest educational group comprised respondents with higher education (42.0%; N = 659), followed by those with secondary education (26.1%; N = 409) and vocational education (21.0%; N = 330).

With regard to region of residence, the Central macroregion was the most strongly represented (21.9%; N = 343), followed by the Southern macroregion (21.0%; N = 330), whereas the smallest proportion of respondents came from the South-Western macroregion (9.9%; N = 155).

Most respondents rated their financial situation as good (71.9%; N = 1127). A poor financial situation was reported by 19.1% (N = 299), while 5.5% (N = 86) described their financial situation as very good. A total of 3.6% (N = 56) declined to answer this question (Table 1).

Among the children included in the analysis, girls accounted for 53.9% (N = 845) and boys for 46.1% (N = 723). The mean age of the children was 10.5 years (SD = 5.2), with a median of 11 years and a range from 0 to 18 years. The largest age group comprised children and adolescents aged 9–18 years (67.3%; N = 1055). Children aged 0–5 years accounted for 25.0% (N = 392) of the analyzed group, while those aged 6–8 years represented 7.7% (N = 121) (Table 2).

### 3.2. Vaccination Status of Children

The vast majority of respondents (95.0%; N = 1489) reported that their child had been vaccinated in accordance with the mandatory vaccination schedule, whereas 5.0% (N = 79) reported non-compliance with mandatory vaccination.

Patterns of recommended vaccine uptake were more heterogeneous. Regular use of recommended vaccines was reported by 35.4% of respondents (N = 555), while 36.8% (N = 577) indicated that such vaccines were used selectively. More than one-quarter of respondents (27.8%; N = 436) reported that the reference child had not received recommended vaccines.

With regard to combination vaccines, 45.1% of parents (N = 707) reported choosing combination formulations, such as the “6-in-1” vaccine. Non-combination vaccines were preferred by 41.8% of respondents (N = 656), whereas 13.1% (N = 205) were unable to indicate a clear preference (Table 3).

### 3.3. Reasons Underlying Decisions to Vaccinate or Not Vaccinate Children

The reasons why respondents chose to comply with mandatory childhood vaccination were analyzed. The results are presented both for the entire study sample (N = 1568) and for respondents who reported vaccinating their child in accordance with the mandatory vaccination schedule (N = 1489) (Table 4).

The most frequently reported reason was the belief that vaccination is necessary for the child’s health. This response was selected by 68.8% (N = 1079) of all respondents and by 72.5% of parents who vaccinated their children. The second most commonly reported reason was the prevention of serious diseases and their complications, indicated by 46.3% of the total sample and 48.8% of respondents who vaccinated their children.

The mandatory nature of vaccination was reported as a reason by 36.9% of all respondents and 38.9% of those who vaccinated their children. Protection of other children and people in the surrounding community was cited by 31.9% of the total sample and 33.6% of vaccinating respondents. Similar proportions were observed for the belief that vaccination is an established and safe preventive measure (29.9% and 31.5%, respectively) and for concern about infectious diseases and their consequences (29.6% and 31.2%, respectively).

Contribution to population immunity was reported as a reason for vaccination by 27.6% of all respondents and 29.1% of those who vaccinated their children. Less frequently reported reasons included trust in recommendations from physicians or other healthcare professionals (20.0% in the total sample and 21.1% among vaccinating respondents) and the belief that the benefits of vaccination outweigh the potential risks (19.1% and 20.1%, respectively). Detailed results are presented in Table 4.

Among parents who did not vaccinate their children according to the mandatory vaccination schedule, the most frequently reported reason was concern about vaccine-related adverse effects (70.9%). More than half of respondents in this group expressed doubts about vaccine safety (50.6%). Other commonly reported reasons included distrust of pharmaceutical companies (41.8%) and concerns about the long-term effects of vaccination (39.2%).

Negative previous experiences with vaccination were reported as a reason for non-vaccination by 30.4% of parents, while 27.8% indicated concern about the number of vaccines administered within a short period of time. The belief that the child had sufficient natural immunity was reported by 24.1% of respondents in this group, whereas 19.0% considered vaccination unnecessary.

Less frequently reported reasons included medical contraindications to vaccination (13.9%) and personal beliefs or worldview (13.9%). Religious beliefs were reported as a reason for non-vaccination by 10.1% of respondents. The least frequently indicated reasons were the influence of other parents or the Internet (7.6%) and other reasons (7.6%) (Table 5).

### 3.4. Vaccine Acceptance

To facilitate interpretation of the findings and enable comparison of the level of agreement with individual statements, mean acceptance scores were calculated, with higher values on the 1–5 scale indicating greater acceptance. The statements were ranked in descending order according to the mean acceptance score. Overall, acceptance of the positive statements was relatively high and showed limited variation. The highest level of agreement was observed for the statement “vaccination has led to the disappearance of many diseases that were once common” (4.08), whereas the lowest was recorded for “vaccinating children is consistent with my religious beliefs” (3.54) (Table 6).

Acceptance scores for negative statements, ranging from 2.68 to 3.10, were clearly lower than those observed for the positive statements. The highest level of agreement was reported for the statement “children receive too many vaccines during the first years of life” (3.10), followed by “vaccines can cause serious adverse effects and complications” (3.08). The lowest level of agreement was observed for the statement suggesting that contracting certain diseases is preferable to vaccination because it provides lifelong immunity (2.68).

### 3.5. Region of Residence, Vaccination Uptake, and Vaccine Acceptance

The analysis of regional variation in vaccination outcomes revealed no statistically significant differences between the macroregions studied. The proportion of parents reporting that their children had been vaccinated in accordance with the mandatory vaccination schedule was very high across all regions, ranging from 93.6% in the Central macroregion to 96.6% in the Northern macroregion (*p* = 0.657). Comparable values were observed in the remaining regions: 94.2% in the South-Western macroregion, 94.5% in the North-Western macroregion, and 95.5% in both the Southern and Eastern macroregions.

For recommended vaccines, the proportion of parents reporting their use ranged from 33.2% in the Northern macroregion to 41.3% in the South-Western macroregion. Selective use of recommended vaccines was reported by 30.3% of respondents in the Eastern macroregion and by 39.7% in the Southern macroregion. The proportion of respondents reporting no use of recommended vaccines ranged from 24.5% in the South-Western macroregion to 31.8% in the Eastern macroregion. These differences were not statistically significant (*p* = 0.438).

No statistically significant regional differences were observed in the use of combination vaccines (*p* = 0.502). Their use was reported by 40.8% of respondents in the Eastern macroregion and by 49.7% in the South-Western macroregion. The proportion of respondents who did not use combination vaccines ranged from 34.8% in the South-Western macroregion to 44.6% in the Eastern macroregion (Table 7).

Before the COVID-19 pandemic, the proportion of respondents retrospectively reporting a positive attitude toward vaccination ranged from 65.9% in the North-Western macroregion to 79.1% in the Southern macroregion, with statistically significant differences between regions (*p* = 0.008). When pre-pandemic and current assessments were compared retrospectively, the largest differences were observed in the Northern (−14.4 percentage points) and Southern (−13.6 percentage points) macroregions. At the time of the study, regional differences in positive vaccination attitudes were less pronounced and were no longer statistically significant (*p* = 0.438). Detailed results are presented in Table 8.

To further assess retrospectively reported differences in attitudes toward vaccination, mean vaccine acceptance scores were calculated on a 1–5 scale for the pre-pandemic period and the time of the study, based on respondents’ retrospective assessments, together with the difference between these scores. The results are presented in Table 9. The color-coding scheme facilitates interpretation of regional variation and retrospectively perceived changes in attitudes toward vaccination following the COVID-19 pandemic.

Before the pandemic, the highest mean acceptance score was observed in the Southern macroregion (4.04), followed by the Northern (3.95) and South-Western (3.88) macroregions. The lowest mean scores were recorded in the Eastern (3.76) and North-Western (3.78) macroregions; however, differences between regions were not statistically significant (*p* = 0.091).

At the time of the study, the highest mean vaccine acceptance score was observed in the Southern macroregion (3.72), followed by the South-Western (3.69) and Central (3.68) macroregions. The lowest values were recorded in the Eastern (3.52) and North-Western (3.55) macroregions, with no statistically significant regional differences (*p* = 0.336).

The largest retrospectively reported decrease in vaccine acceptance was observed in the Northern macroregion, where the mean score difference between the pre-pandemic period and the time of the study was −0.37 points, followed by the Southern macroregion, with a difference of −0.32 points. Smaller retrospectively reported decreases were observed in the Eastern (−0.25), North-Western (−0.23), South-Western (−0.19), and Central (−0.16) macroregions. Regional differences in the magnitude of retrospectively reported differences were statistically significant (*p* = 0.013) (Table 9).

## 4. Discussion

In this nationwide sample of parents of children and adolescents, self-reported compliance with mandatory vaccination was high; however, this was not accompanied by uniformly high vaccine acceptance. Recommended vaccines were used less frequently than mandatory vaccines, and a substantial proportion of respondents reported selective uptake of recommended vaccinations. Concerns regarding vaccine safety, adverse effects, and the number of vaccines administered during early childhood also remained evident. No statistically significant macroregional differences were observed in self-reported uptake of mandatory, recommended, or combination vaccines. Across all macroregions, respondents retrospectively rated their current attitudes toward vaccination as less positive than during the pre-COVID-19 period; however, this finding should be interpreted with particular caution.

The coexistence of high compliance with mandatory vaccination, selective uptake of recommended vaccines, and persistent safety concerns supports the concept of vaccine hesitancy as a continuum rather than a phenomenon equivalent to complete vaccine refusal [2,3]. This distinction is important because, even when current vaccination uptake remains high, declining confidence may contribute to delayed vaccination, selective acceptance, or future refusal, thereby potentially reducing the health benefits achieved through childhood immunization programs [1,4].

The level of self-reported compliance with mandatory vaccination observed in the present study was comparable to that reported in a nationwide study by Furman et al., in which 97.7% of parents declared that their children were vaccinated according to the national vaccination program [7]. The 95.0% observed in the present study is also broadly consistent with administrative data indicating high, although incomplete, vaccination coverage among children and adolescents in Poland [21]. At the same time, national surveillance data indicate an increasing number of cases of avoidance of mandatory vaccination in recent years [22,23]. These administrative indicators are not directly comparable with the present findings because they are based on different definitions, age groups, and reporting systems; however, they provide relevant context for interpreting the high but not universal level of self-reported compliance observed in our study.

The strongest motivations for compliance with mandatory vaccination were the belief that vaccination is necessary for the child’s health and its role in preventing serious diseases and their complications. At the same time, 38.9% of parents who vaccinated their children indicated the mandatory nature of vaccination as one of the reasons for their decision. This finding indicates that the legal status of mandatory vaccination was one of several factors considered by some parents in their vaccination decisions. However, the present study does not allow conclusions to be drawn regarding how these parents would have behaved in the absence of the mandatory vaccination requirement. In another Polish study by Furman et al., 8.5% of parents who complied with vaccination recommendations stated that they would discontinue vaccination if the legal requirement were removed [7]. Taken together, these findings suggest that vaccination behavior and underlying vaccine acceptance may not always fully overlap.

The reasons most frequently reported for non-vaccination were predominantly related to safety concerns. Among parents in this group, 70.9% expressed concern about adverse effects, 50.6% reported doubts about vaccine safety, 41.8% reported distrust of pharmaceutical companies, and 39.2% expressed concern about long-term effects. A similar pattern emerged from responses to the attitudinal statements: parents generally rated the importance and effectiveness of vaccination highly, whereas ratings related to safety and personal confidence were lower. Among the negative statements, the highest levels of agreement concerned the perception that children receive too many vaccines in early childhood and that vaccines may cause serious adverse effects. These findings suggest that the weakest component of parental attitudes was not rejection of the concept of prevention itself, but rather confidence in vaccine safety and the institutions associated with vaccination. This interpretation is consistent with previous Polish studies in which perceptions of vaccine safety were associated with sources of information, previous experiences, perceived adverse events following immunization, and the quality of medical assessment before vaccination [8,9]. A recent review of Polish evidence likewise identified concerns about adverse effects and doubts regarding vaccine effectiveness as major barriers to vaccination [12]. In addition, a post-COVID-19 study identified dissatisfaction with medical communication, lack of confidence in selected components of the vaccination schedule, and individualized assessment of benefits and risks [15].

The discrepancy between mandatory and recommended vaccination deserves particular attention. Only 35.4% of parents reported regular use of recommended vaccines, 36.8% reported selective use, and 27.8% reported no use at all. This distribution reflects the selective dimension of vaccine hesitancy, which may relate to a specific vaccine or the child’s age rather than to rejection of the vaccination program as a whole [4]. With regard to combination vaccines, 41.8% of respondents did not choose such vaccines, while 13.1% were unable to indicate a preference. The absence of a clear predominance of either option, together with the proportion of “I do not know” responses, highlights the need for clear information regarding the number of injections, vaccination schedules, safety, and financing. The lower uptake of recommended compared with mandatory vaccines further indicates that parental vaccination decisions are selective. Such selectivity may reflect differences in perceived disease risk, perceived necessity of a particular vaccine, cost, accessibility, the child’s age, or the nature of the recommendation provided by a healthcare professional. Because the present study did not examine all of these mechanisms in detail or analyze each recommended vaccine separately, the observed pattern cannot be attributed to a single factor. Nevertheless, it provides an important basis for further research focusing on individual vaccines, financing, accessibility, and the quality of recommendations communicated to parents.

One of the notable findings of the present study was the retrospectively reported decrease in support for vaccination following the COVID-19 pandemic across all macroregions. The largest retrospectively reported decreases were identified in the Northern and Southern macroregions, and regional differences in the magnitude of these reported differences were statistically significant. However, this pattern is not universal. In a Canadian study, some parents perceived routine childhood vaccinations as more important after the onset of the pandemic, and confidence in their safety increased [13]. A study conducted in the United States found no overall increase in hesitancy toward routine childhood vaccination, although changes in confidence in vaccine-related information and differences between parental subgroups were observed [14].

In contrast, a comparative study conducted in the United Kingdom and Israel identified, among some parents, a shift toward non-vaccination and increased concerns regarding adverse effects and trust [24]. The findings of the present study are more consistent with this latter pattern and with observations reported in Polish parents by Janiak et al. [15]. Differences between countries suggest that the pandemic did not act as a single, uniformly directional determinant of vaccination attitudes. Cross-national differences in vaccination attitudes should also be interpreted in the context of the sociodemographic composition of the populations studied. Previous European research has shown that vaccine confidence and vaccination attitudes may be associated with factors such as age, sex, and educational attainment, while differences between countries may additionally reflect healthcare-system characteristics and the sources of information and professional advice available to parents [6]. Other European analyses have similarly linked vaccination attitudes to age, gender, educational attainment, and trust in different information sources. However, the direction and strength of these associations are not consistent across countries, indicating that sociodemographic determinants of vaccine hesitancy are context-dependent rather than universal [25]. Therefore, cross-national differences in vaccination attitudes should be interpreted cautiously and should not be attributed to any single sociodemographic factor. The influence of the COVID-19 pandemic on vaccination attitudes, as reflected in retrospectively reported changes, may also have been modified by pre-existing levels of trust, experiences with the healthcare system, politicization of the vaccination debate, and exposure to misinformation. Importantly, the observed change in attitudes should be interpreted as a retrospectively reported change rather than as a directly measured longitudinal effect. In 2026, respondents were asked to recall their attitudes from the pre-pandemic period; consequently, their responses may have been affected by recall bias or by reconstruction of previous beliefs in light of subsequent experiences. Nevertheless, these findings provide insight into how parents currently perceive changes in their own attitudes toward vaccination.

The absence of statistically significant macroregional differences in current uptake of mandatory, recommended, and combination vaccines suggests limited variation at the level of the six broad macroregions analyzed. However, these broad geographical categories may mask meaningful differences at the voivodeship or local level and should not be interpreted as evidence of regional uniformity. Similarly, although differences in positive attitudes toward vaccination were statistically significant between macroregions for the retrospectively assessed pre-pandemic period, but not at the time of the study, this pattern should be interpreted cautiously and does not necessarily indicate convergence of vaccination attitudes within or across individual regions. The apparent stability of vaccination behavior despite retrospectively reported lower current acceptance may be relevant from a preventive perspective, as previous evidence indicates that parental vaccine hesitancy increases the risk of delayed or missed vaccination, which may be particularly important in light of reports showing deterioration in selected indicators of vaccination program implementation [4,22].

The present findings identify several areas that may warrant attention in future vaccination-related communication and intervention efforts, particularly concerns regarding vaccine safety, adverse effects, and the selective use of recommended vaccines. However, the present study did not evaluate the effectiveness of any specific communication or behavioral intervention. Therefore, particular strategies cannot be recommended on the basis of these findings alone. Previous studies suggest that approaches such as transparent communication about vaccine safety and adverse events, discussion of disease risks and the benefits of vaccination, improved access to reliable information, reminder systems, and support from healthcare professionals may be relevant in addressing some of the barriers identified in this study [8,10,26]. These approaches should therefore be regarded as literature-informed options for future research and practice rather than interventions whose effectiveness was demonstrated by the present study.

### Strengths and Limitations

Several strengths of this study should be highlighted. First, the study included a large nationwide sample of 1568 parents of children and adolescents aged 0–18 years. Second, a stratified quota sampling approach was used to reflect key sociodemographic characteristics of the target population. Third, respondents from all six Polish macroregions were included, allowing regional patterns in vaccination uptake and attitudes to be examined. Finally, the study simultaneously assessed self-reported compliance with mandatory vaccination, use of recommended vaccines, reasons underlying vaccination-related decisions, and parental attitudes toward vaccination, providing a broad overview of parental vaccination behaviors and perceptions in Poland.

Despite these strengths, several limitations should be considered when interpreting the findings of this study. The cross-sectional design does not allow causal relationships to be established. Vaccination status, use of recommended vaccines, and attitudes toward vaccination were based on parental self-report and were not independently verified against medical or vaccination records; therefore, recall and social desirability bias cannot be excluded.

Particular caution is required when interpreting the reported change in attitudes toward vaccination since the COVID-19 pandemic. Pre-pandemic attitudes were assessed retrospectively in 2026 rather than measured prospectively in the same individuals before and after the pandemic. Consequently, the observed differences should be interpreted as perceived changes in attitudes and may be affected by recall bias and by respondents’ current beliefs and experiences. In addition, only 79 respondents reported that their child was not vaccinated according to the mandatory vaccination schedule, limiting the precision of analyses concerning reasons for non-vaccination and precluding more detailed subgroup comparisons. Regional analyses were conducted at the level of six broad macroregions and may therefore have obscured differences occurring at the local level.

Another limitation concerns the assessment of the use of recommended vaccines. This was based on a general question regarding the use of recommended vaccinations rather than on separate assessments of individual vaccines. In particular, the response category “In selected cases” may have included a heterogeneous group of parents who accepted some recommended vaccines while declining others for different reasons, such as the type of vaccine, the child’s age, vaccination costs, vaccine availability, healthcare provider recommendations, or the perceived risk of the disease. Therefore, the findings should be interpreted as reflecting parents’ general approach toward recommended vaccinations rather than the uptake of specific recommended vaccines. Future studies should assess individual recommended vaccines separately and examine vaccine-specific factors influencing parental decision-making. These limitations should be taken into account when interpreting the observed patterns and designing future longitudinal and vaccine-specific studies.

## 5. Conclusions

In this nationwide sample of parents, self-reported compliance with mandatory childhood vaccination was high, whereas recommended vaccines were used less frequently and often selectively. High uptake of mandatory vaccination coexisted with persistent concerns regarding vaccine safety, adverse effects, and potential long-term consequences, indicating that compliance with mandatory vaccination should not be regarded as a complete indicator of sustained confidence in vaccination. No significant differences were identified across the six Polish macroregions in the reported uptake of mandatory, recommended, or combination vaccines. Respondents retrospectively assessed their current attitudes toward vaccination as less positive than during the pre-COVID-19 period; however, this finding should be confirmed in longitudinal studies. Overall, the findings identify vaccine safety, adverse events following immunization, and the use of recommended vaccines as important areas for future educational and communication efforts. Transparent, evidence-based communication by healthcare professionals may be relevant in supporting vaccine confidence and informed vaccination decisions; however, the effectiveness of specific communication strategies was not assessed in the present study.

These findings may support the development of more targeted vaccination communication and education strategies addressed to parents with different levels of vaccine acceptance. Future research should focus on longitudinal assessment of changes in parental attitudes and on identifying modifiable determinants of vaccine hesitancy. Further studies should also evaluate the effectiveness of interventions aimed at improving confidence in both mandatory and recommended childhood vaccinations.

## Figures and Tables

**Table 1:** Sociodemographic Characteristics of the Study Population.

Parameter		Total (1568)
Age [years]	Mean (SD)	42.1 (8.4)
	Median	42
	Min-max	19–66
Sex	Female	783 (49.9%)
	Male	785 (50.1%)
Place of residence	Rural area	652 (41.6%)
	City up to 50 th. inhab.	304 (19.4%)
	City 50–100 th. inhab.	186 (11.9%)
	City 100–500 th. inhab.	239 (15.2%)
	City over 500 th. inhab.	187 (11.9%)
Marital status	Single	197 (12.6%)
	In relationship	1371 (87.4%)
Education level	Primary education	49 (3.1%)
	Vocational education	330 (21.0%)
	Secondary education	409 (26.1%)
	Post-secondary education	121 (7.7%)
	Higher education	659 (42.0%)
Region of residence	South-Western	155 (9.9%)
	North-Western	238 (15.2%)
	Central	343 (21.9%)
	Southern	330 (21.0%)
	Eastern	267 (17.0%)
	Northern	235 (15.0%)
Financial situation	Very good	86 (5.5%)
	Good	1127 (71.9%)
	Bad	299 (19.1%)
	Refused to answer	56 (3.6%)

More than half of the respondents reported having one child (52.7%; N = 827), while 36.2% (N = 568) had two children. The table shows that 8.4% (N = 132) of respondents had three children, whereas 2.6% (N = 41) had four or more children.

**Table 2:** Number of Children in the Family and Characteristics of the Randomly Selected Reference Child.

Parameter		Total (1568)
Number of children	1	827 (52.7%)
	2	568 (36.2%)
	3	132 (8.4%)
	4 and more	41 (2.6%)
Child’s sex	female	845 (53.9%)
	male	723 (46.1%)
Child’s age group [years]	0–5	392 (25.0%)
	6–8	121 (7.7%)
	9–18	1055 (67.3%)
Age [years]	Mean (SD)	10.5 (5.2)
	Median	11
	Min-max	0–18

**Table 3:** Self-Reported Childhood Vaccination Status and Use of Recommended and Combination Vaccines.

Response	N	%
**Vaccination in Accordance with the Mandatory Vaccination Schedule**		
Yes	1489	95.0%
No	79	5.0%
Use of recommended vaccines		
Yes	555	35.4%
In selected cases	577	36.8%
No	436	27.8%
Use of combination vaccines		
I prefer non-combination vaccines	656	41.8%
I choose combination vaccines (e.g., 6-in-1)	707	45.1%
I do not know	205	13.1%

**Table 4:** Reasons for Compliance with Mandatory Childhood Vaccination.

Reasons for Mandatory Childhood Vaccination		In the Total Study Population (N = 1568)	Among Respondents Who Vaccinated Their Children (N = 1489)
	N	% ^1^	% ^1^
Vaccination is necessary for the child’s health	1079	68.8%	72.5%
Prevention of serious diseases and their complications	726	46.3%	48.8%
Because vaccination is mandatory	579	36.9%	38.9%
Protection of other children and people in the surrounding community	500	31.9%	33.6%
Vaccination is an established and safe preventive measure	469	29.9%	31.5%
Concern about infectious diseases and their consequences	464	29.6%	31.2%
Contribution to population immunity	433	27.6%	29.1%
Trust in recommendations from physicians or other healthcare professionals	314	20.0%	21.1%
The benefits of vaccination outweigh the potential risks	299	19.1%	20.1%
Avoidance of formal difficulties (e.g., in kindergarten or school)	129	8.2%	8.7%
Other	12	0.8%	0.8%

^1^ Results do not sum to 100% because respondents could select more than one response option.

**Table 5:** Reasons for Forgoing Mandatory Childhood Vaccination.

Reasons for Non-Compliance with Mandatory Childhood Vaccination		In the Total Study Population (N = 1568)	Among Respondents Who Did Not Vaccinate Their Children (N = 79)
	N	% ^1^	% ^1^
Vaccine-related adverse effects	56	3.6%	70.9%
Concerns about vaccine safety	40	2.6%	50.6%
Distrust of pharmaceutical companies	33	2.1%	41.8%
Concerns about the long-term effects of vaccination	31	2.0%	39.2%
Negative previous experiences with vaccination	24	1.5%	30.4%
Too many vaccines administered within a short period of time	22	1.4%	27.8%
The child has sufficient natural immunity	19	1.2%	24.1%
Vaccination is unnecessary	15	1.0%	19.0%
Medical contraindications to vaccination	11	0.7%	13.9%
Conflicting information about vaccination	11	0.7%	13.9%
Personal beliefs or worldview	11	0.7%	13.9%
Religious beliefs	8	0.5%	10.1%
Influence of other parents or the Internet	6	0.4%	7.6%
Other	6	0.4%	7.6%

^1^ Results do not sum to 100% because respondents could select more than one response option.

**Table 6:** Mean Acceptance Scores for Positive and Negative Statements Regarding Vaccination.

**Positive Statements Regarding Vaccination**	$\bar{x}$
Vaccination has led to the disappearance of many diseases that were once common	4.08 ^1^
Vaccines are important	4.05
Vaccination provides the best protection for children against serious diseases	3.93
Vaccinating children provides more benefits than risks	3.91
Vaccination protects not only the vaccinated individual but also other people	3.91
Vaccines are effective	3.90
Vaccines for children are safe	3.66
I have confidence in vaccines	3.64
Vaccinating children is consistent with my religious beliefs	3.54
**Negative Statements Regarding Vaccination**	$\bar{x}$
Children receive too many vaccines during the first years of life	3.10
Vaccines can cause serious adverse effects and complications	3.08
There are too many vaccines in the vaccination schedule	3.07
Vaccination is promoted by pharmaceutical companies	2.97
Vaccination may weaken a child’s natural immunity	2.82
Vaccines may lead to developmental disorders such as autism	2.73
It is better to contract certain diseases and acquire lifelong immunity than to be vaccinated	2.68

^1^ Scores were calculated using the following coding: 1 = no acceptance (no), …, 5 = acceptance (yes).

**Table 7:** Childhood Vaccination Uptake by Respondents’ Region of Residence.

Vaccination Uptake		Region											
		South-Western		North-Western		Central		Southern		Eastern		Northern	
		N	%	N	%	N	%	N	%	N	%	N	%
Vaccination in accordance with the mandatory vaccination schedule (*p* = 0.657)	Yes	146	94.2	225	94.5	321	93.6	315	95.5	255	95.5	227	96.6
	No	9	5.8	13	5.5	22	6.4	15	4.5	12	4.5	8	3.4
Use of recommended vaccines (*p* = 0.438)	Yes	64	41.3	80	33.6	122	35.6	110	33.3	101	37.8	78	33.2
	In selected cases	53	34.2	91	38.2	131	38.2	131	39.7	81	30.3	90	38.3
	No	38	24.5	67	28.2	90	26.2	89	27.0	85	31.8	67	28.5
Use of combination vaccines (*p* = 0.502)	Yes	77	49.7	103	43.3	169	49.3	141	42.7	109	40.8	108	46.0
	No	54	34.8	104	43.7	135	39.4	147	44.5	119	44.6	97	41.3
	I do not know	24	15.5	31	13.0	39	11.4	42	12.7	39	14.6	30	12.8

**Table 8:** Respondents’ Attitudes Toward Vaccination Before the COVID-19 Pandemic and at Present, organized by Region of Residence.

Attitudes Toward Vaccination Before and After the COVID-19 Pandemic		Region											
		South-Western		North-Western		Central		Southern		Eastern		Northern	
		N	%	N	%	N	%	N	%	N	%	N	%
Positive attitude toward vaccination before the COVID-19 pandemic (*p* = 0.008)	Yes	48	31.0	81	34.0	104	30.3	117	35.5	83	31.1	76	32.3
	Rather yes	64	41.3	76	31.9	144	42.0	144	43.6	95	35.6	98	41.7
	I do not know	26	16.8	49	20.6	51	14.9	42	12.7	47	17.6	43	18.3
	Rather no	10	6.5	11	4.6	25	7.3	20	6.1	27	10.1	9	3.8
	No	7	4.5	21	8.8	19	5.5	7	2.1	15	5.6	9	3.8
	Yes	44	28.4	67	28.2	106	30.9	91	27.6	67	25.1	53	22.6
Current positive attitude toward vaccination (*p* = 0.438)	Rather yes	58	37.4	74	31.1	106	30.9	125	37.9	91	34.1	87	37.0
	I do not know	27	17.4	45	18.9	73	21.3	62	18.8	50	18.7	54	23.0
	Rather no	13	8.4	27	11.3	32	9.3	35	10.6	31	11.6	26	11.1
	No	13	8.4	25	10.5	26	7.6	17	5.2	28	10.5	15	6.4

**Table 9:** Regional variation in mean vaccine acceptance scores before the COVID-19 pandemic and at present.

Region	Mean Vaccine Acceptance Score		
	Before the Pandemic	At the Time of the Study	Difference
South-Western	3.88	3.69	−0.19
North-Western	3.78	3.55	−0.23
Central	3.84	3.68	−0.16
Southern	4.04	3.72	−0.32
Eastern	3.76	3.52	−0.25
Northern	3.95	3.58	−0.37
*p*	0.091	0.336	0.013 *

Mean scores were calculated using the following coding: 1 = no, 2 = rather no, 3 = I do not know, 4 = rather yes, and 5 = yes (higher values indicate greater support for vaccination). *p* = probability values calculated using the Kruskal–Wallis test; * statistically significant.

## Data Availability

Data can be obtained by contacting the corresponding author.

## Supplementary Materials

The following supporting information can be downloaded at: https://www.mdpi.com/article/10.3390/vaccines14100906/s1, Supplementary File S1: Parental Attitudes toward Childhood and Adolescent Vaccination in Poland after the COVID-19 Pandemic. Questionnaire used in the study, including the original Polish-language version and its English translation.
