# Supplementary material for: Parental Attitudes Toward Childhood and Adolescent Vaccination in Poland in 2026: Vaccination Uptake and Retrospectively Reported Changes Since the Pre-COVID-19 Period
Source: Vaccines (Basel). 2026 Oct 17;14(10):906. doi: 10.3390/vaccines14100906 (PMC13709576; doi:10.3390/vaccines14100906)
Supplement: Supplementary file 1 [file vaccines-14-00906-s001.zip › vaccines-4565542-supplementary.pdf]

File S1

Postawy rodziców dzieci i młodzieży wobec szczepień ochronnych w Polsce po pandemii COVID-19

Szanowna/y Pani/y

Dziękujemy za wypełnienie poniższej ankiety. Celem badania jest ocena postaw rodziców dzieci i młodzieży wobec szczepień ochronnych po pandemii COVID-19. Ankieta jest anonimowa i będzie wykorzystana wyłącznie do celów badawczych.

I. <u>INFORMACJE DEMOGRAFICZNE i SPOŁECZNE</u>

1. Płeć:
   a) kobieta
   b) mężczyzna

2. Wiek w latach:
   ……
   …………………………………………………

3. Wykształcenie
   a) podstawowe
   b) zawodowe
   c) średnie
   d) policelane
   e) wyższe

4. Stan cywilny
   a) wolna/y
   b) w związku

5. Miejsce zamieszkania (miasto/wieś)
   a) wieś
   b) miasto do 50 tys. mieszkańców
   c) miasto powyżej 50 do 100 000 mieszkańców
   d) miasto powyżej 100 000 mieszkańców

6. Miejsce zamieszkania (województwo)
   a) dolnośląskie
   b) kujawsko-pomorskie
   c) lubelskie
   d) lubuskie
   e) łódzkie
   f) małopolskie
   g) mazowieckie
   h) opolskie
   i) podkarpackie
   j) podlaskie
   k) pomorskie
   l) śląskie
   m) świętokrzyskie
   n) warmińsko-mazurskie
   o) wielkopolskie
   p) zachodniopomorskie

7. Sytuacja finansowa
   a) bardzo dobra

b) dobra
c) zła

8. Liczba dzieci
a) 1
b) 2
c) 3
d) 4
e) więcej niż 4
f) nie mam dzieci poniżej 18 lat

9. Płeć dziecka/dzieci
- dziecko nr 1 - …….
- dziecko nr 2 - ……….
- dziecko nr 3 - ……..
- dziecko nr 4 - ……..
- dziecko nr 5 - ………

10. Wiek dziecka (w latach):

- dziecko nr 1 - …….
- dziecko nr 2 - ……….
- dziecko nr 3 - ……..
- dziecko nr 4 - ……..
- dziecko nr 5 - ………

II. INFORMACJE O SZCZEPIENIU DZIECKA/DZIECI

11. Czy szczepi Pan/Pani swoje dziecko/dzieci zgodnie z obowiązującym kalendarzem szczepień?
a) tak
b) nie - *proszę przejść do pytania nr 13*

12. Dlaczego zdecydował/a się Pan/Pani na szczepienie swojego dziecka? (możliwość wyboru kilku odpowiedzi)
a) uważam, że szczepienia są konieczne dla zdrowia dziecka
b) ponieważ są obowiązkowe
c) zależy mi na ochronie innych dzieci i osób w otoczeniu
d) zaufałem/am rekomendacjom lekarza lub personelu medycznego
e) chcę zapobiec poważnym chorobom i ich powikłaniom
f) szczepienia są sprawdzoną i bezpieczną metodą profilaktyki
g) obawiam się chorób zakaźnych i ich konsekwencji

h) szczepienia zwiększają odporność populacyjną (tzw. odporność zbiorową)
i) chciałem/am uniknąć problemów formalnych (np. w przedszkolu, szkole)
j) uznałem/am, że korzyści ze szczepień przewyższają ewentualne ryzyko
k) inne

Proszę przejść do pytania nr 14

13. Dlaczego nie zdecydował/a się Pan/Pani na szczepienie swojego dziecka? (możliwość wyboru kilku odpowiedzi)
    a) obawiam się skutków ubocznych szczepionek
    b) uważam, że szczepienia są zbędne
    c) mam wątpliwości co do bezpieczeństwa szczepionek
    d) obawiam się długoterminowych skutków szczepień
    e) uważam, że dziecko ma wystarczającą odporność naturalną
    f) miałem/am negatywne doświadczenia związane ze szczepieniami (u dziecka i/lub w rodzinie)
    g) moje dziecko ma przeciwwskazania zdrowotne do szczepień
    h) otrzymałem/am sprzeczne informacje na temat szczepień
    i) nie ufam firmom farmaceutycznym produkującym szczepionki
    j) decyzja była wynikiem własnych przekonań lub światopoglądu
    k) obawiam się zbyt dużej liczby szczepień w krótkim czasie
    l) szczepienia nie są zgodne z moimi przekonaniami religijnymi
    m) decyzja została podjęta po konsultacji z innymi rodzicami i/lub pod wpływem źródeł internetowych
    n) inne

14. Czy decyduje się Pan/Pani na zalecane szczepienia dzieci, które **<u>nie</u>** są obowiązkowe (tzw. szczepienia zalecane"?
    a) tak – *proszę przejść do pytania 16*
    b) nie
    c) tylko w niektórych przypadkach

15. Z jakiego powodu nie decyduje się Pan/Pani na szczepienia zalecane dla swojego dziecka? (można wybrać kilka odpowiedzi)
    a) obawiam się skutków ubocznych szczepionek
    b) uważam, że szczepienia zalecane są zbędne
    c) mam wątpliwości co do bezpieczeństwa szczepionek
    d) dziecko jest zdrowe i nie ma potrzeby dodatkowych szczepień
    e) szczepionki zlecane zazwyczaj są zbyt drogie
    f) dziecko ma dużo szczepień obowiązkowych i niepotrzebne są mu szczepienia zalecane
    g) obawiam się długoterminowych skutków szczepień
    h) uważam, że odporność naturalna dziecka jest wystarczająca
    i) otrzymuję sprzeczne informacje na temat szczepień zalecanych
    j) brak jest wystarczających informacji od personelu medycznego na ten temat
    k) nie ufam producentom szczepionek
    l) inne

16. Czy decyduje się Pan/Pani na stosowanie szczepionek oferowanych przez służbę zdrowia, czy woli Pan/Pani szczepionki łączone (np. 6 w 1), aby uniknąć wielokrotnych ukłuć?
    a) decyduję się na szczepionki łączone (np. 6 w 1)
    b) preferuję szczepionki oferowane przez służbę zdrowia, nawet jeśli oznacza to więcej ukłuć
    c) nie wiem

III. <u>POSTAWY WOBEC SZCZEPIEŃ DZIECI</u>

17. Czy uważa Pan/i, że szczepienia stanowią najlepszą ochronę dla dzieci przed ciężkimi chorobami:
    a) tak, zdecydowanie się zgadzam
    b) raczej się zgadzam
    c) nie mam jednoznacznej opinii na ten temat
    d) raczej się nie zgadzam
    e) zdecydowanie się nie zgadzam się

18. Czy posiada Pan/i zaufanie do szczepień?

a) mam duże zaufanie
b) raczej mam zaufanie
c) nie mam opinii na ten temat
d) raczej nie mam zaufania
e) nie mam zaufania

19. Czy uważa Pan/i, że szczepionki dla dzieci są bezpieczne?
   a) całkowicie bezpieczne
   b) raczej bezpieczne
   c) ryzykowne
   d) bardzo ryzykowne
   e) nie mam zadania

20. Czy uważa Pan/i, że szczepionki są ważne?
   a) bardzo ważne
   b) raczej ważne
   c) nie mam opinii na ten temat
   d) nie tak ważne, jak im się przypisuje znaczenie
   e) zdecydowanie nieważne

21. Czy uważa Pan/i, że szczepionki są skuteczne?
   a) są bardzo skuteczne
   b) są skuteczne
   c) nie mam opinii na ten temat
   d) raczej nie są skuteczne
   e) zdecydowanie nie są skuteczne

22. Czy uważa Pan/i, że dzięki szczepieniom ochronnym, wiele niebezpiecznych chorób, które kiedyś były powszechne, dziś niemal zniknęły?
   a) tak, zdecydowanie się zgadzam
   b) raczej się zgadzam
   c) nie mam jednoznacznej opinii na ten temat
   d) raczej się nie zgadzam
   e) zdecydowanie się nie zgadzam się

23. Czy uważa Pan/i, że zasadniczo, szczepienie dzieci przynosi więcej korzyści niż ryzyka?
   a) tak, zdecydowanie się zgadzam
   b) raczej się zgadzam
   c) nie mam jednoznacznej opinii na ten temat.
   d) raczej się nie zgadzam
   e) zdecydowanie się nie zgadzam się

24. Proszę odnieść się do poniższego stwierdzenia:
„Szczepienie dzieci jest zgodne z moimi przekonaniami religijnymi”:
   a) tak, zdecydowanie się zgadzam
   b) raczej się zgadzam
   c) nie mam jednoznacznej opinii na ten temat
   d) raczej się nie zgadzam
   e) zdecydowanie się nie zgadzam się

25. Proszę odnieść się do poniższego stwierdzenia:
„Szczepienie nie tylko chroni osobę, która je przyjmuje, ale także innych ludzi”:
   a) tak, zdecydowanie się zgadzam
   b) raczej się zgadzam
   c) nie mam jednoznacznej opinii na ten temat.
   d) raczej się nie zgadzam
   e) zdecydowanie się nie zgadzam się

26. Proszę odnieść się do poniższego stwierdzenia:

„Szczepionki dla dzieci mogą prowadzić do poważnych zaburzeń rozwojowych, takich jak autyzm”.

a) tak, zdecydowanie się zgadzam
b) raczej się zgadzam
c) nie mam jednoznacznej opinii na ten temat.
d) raczej się nie zgadzam
e) zdecydowanie się nie zgadzam się

27. Proszę odnieść się do poniższego stwierdzenia:
„Szczepienia mogą osłabiać naturalną odporność dziecka”.

a) tak, zdecydowanie się zgadzam
b) raczej się zgadzam
c) nie mam jednoznacznej opinii na ten temat.
d) raczej się nie zgadzam
e) zdecydowanie się nie zgadzam się

28. Proszę odnieść się do poniższego stwierdzenia:
„Uważam, że lepiej pozwolić dziecku przejść przez chorobę zakaźną, taką jak odra czy ospa, niż szczepić, bo po kilku dniach choroby dziecko zyskuje naturalną odporność na całe życie”.

a) tak, zdecydowanie się zgadzam
b) raczej się zgadzam
c) nie mam jednoznacznej opinii na ten temat
d) raczej się nie zgadzam
e) zdecydowanie się nie zgadzam się

29. Proszę odnieść się do poniższego stwierdzenia:
„Szczepionki dla dzieci mogą powodować poważne skutki uboczne i powikłania”:

a) tak, zdecydowanie się zgadzam
b) raczej się zgadzam
c) nie mam jednoznacznej opinii na ten temat.
d) raczej się nie zgadzam
e) zdecydowanie się nie zgadzam się

30. Proszę odnieść się do poniższego stwierdzenia:
„W początkowych latach życia dzieci otrzymują zbyt wiele szczepionek”:

a) tak, zdecydowanie się zgadzam
b) raczej się zgadzam
c) nie mam jednoznacznej opinii na ten temat.
d) raczej się nie zgadzam
e) zdecydowanie się nie zgadzam się

31. Proszę odnieść się do poniższego stwierdzenia:
„W obowiązującym kalendarzu szczepień jest ich zbyt wiele – liczba szczepień powinna być mniejsza”:

a) tak, zdecydowanie się zgadzam
b) raczej się zgadzam
c) nie mam jednoznacznej opinii na ten temat.
d) raczej się nie zgadzam
e) zdecydowanie się nie zgadzam się

32. Proszę odnieść się do poniższego stwierdzenia:
„Szczepienia są promowane nie z powodu ich rzeczywistej potrzeby, lecz dlatego, że jest to korzystne dla koncernów farmaceutycznych”:

a) tak, zdecydowanie się zgadzam
b) raczej się zgadzam
c) nie mam jednoznacznej opinii na ten temat.
d) raczej się nie zgadzam
e) zdecydowanie się nie zgadzam się

IV. Postawy wobec pandemii i jej wpływ na decyzje wobec szczepień

33. Czy przed pandemią był Pan/i zwolennikiem szczepień?
    a) tak, byłem zwolennikiem wszystkich szczepień.
    b) raczej tak
    c) nie mam jednoznacznej opinii na ten temat
    d) raczej nie
    e) nie, nie byłem zwolennikiem szczepień.

34. Czy aktualnie jest Pan/i zwolennikiem szczepień?
    a) tak, jestem zwolennikiem szczepień.
    b) raczej tak
    c) nie mam jednoznacznej opinii.
    d) raczej nie
    e) nie, nie jestem zwolennikiem szczepień.

35. Czy pandemia COVID-19 wpłynęła na Pana/i postawy wobec szczepień?
    a) tak, zdecydowanie
    b) tak, do pewnego stopnia
    c) nie mam jednoznacznej opinii *(proszę przejść do pytania nr 37)*
    d) raczej nie (*proszę przejść do pytania nr 37)*
    e) nie, nie miało to wpływu (*proszę przejść do pytania nr 37)*

36. W jaki sposób pandemia COVID-19 wpłynęła na Pana/i postawy wobec szczepień?
    a) pandemia utwierdziła mnie w przekonaniu, że szczepienia są niezbędne
    b) pandemia spowodowała, że stałem/łam się zwolennikiem szczepień
    c) pandemia utwierdziła mnie w przekonaniu, że szczepienia są zbędne
    d) pandemia spowodowała, że stałem/łam się przeciwnikiem szczepień
    e) pandemia nie miała wpływu na mój stosunek do szczepień

37. Czy Pana/i ocena roli szczepień w walce z pandemią COVID-19 zmieniła się w wyniku doświadczeń związanych z pandemią?
    a) zdecydowanie tak
    b) raczej tak
    c) raczej nie
    d) zdecydowanie nie
    e) nie mogę ocenić / trudno powiedzieć

38. Czy Pana/i zaufanie do systemu ochrony zdrowia zwiększyło się lub zmniejszyło się w wyniku pandemii COVID-19?
    a) zdecydowanie zwiększyło
    b) raczej zwiększyło
    c) nie miało to wpływu
    d) raczej zmniejszyło
    e) zdecydowanie zmniejszyło

39. Czy przyjął Pan/i szczepionkę przeciw COVID-19?
    a) tak, w sytuacji pandemii uważałem to za słuszne – *proszę przejść do pytania 41*
    b) tak, zrobiłem/łam to tylko dlatego, gdyż w innym wypadku nie mógłbym/mogłabym wykonywać pracy zawodowej - *proszę przejść do pytania 41*
    c) nie

40. Z jakiego powodu nie zaszczepił/a się Pan/i przeciw COVID-19? (możliwość wyboru kilku odpowiedzi)
    a) obawy dotyczące bezpieczeństwa szczepionek
    b) brak przekonania o skuteczności szczepień
    c) problemy z dostępnością szczepionek
    d) opór ideologiczny lub religijny
    e) niezrozumienie lub brak wiedzy na temat procesu szczepienia
    f) niskie zaufanie do instytucji publicznych

g) generalnie nie ufam szczepionkom
h) inne

41. Czy doświadczenia związane z pandemią (np. obawy o zdrowie, trudności związane z leczeniem pacjentów) skłoniły Pana/ią do bardziej pozytywnego nastawienia do szczepień?
a) zdecydowanie tak
b) raczej tak, w pewnym stopniu
c) nie wiem, trudno powiedzieć
d) raczej nie
e) nie, nie miało to wpływu

V. EDUKACJA I INFORAMCJA O SZCZEPIENIACH

42. Które z poniższych źródeł informacji mają na Pana/ia największy wpływ w kształtowaniu Pana/i postaw i opinii na temat szczepień:
a) pracownicy służby zdrowia (np. lekarze, pielęgniarki)
b) media (telewizja, radio, prasa)
c) internet (strony internetowe, fora dyskusyjne)
d) wykłady akademickie
e) opinie przyjaciół i rodziny

43. Czy uważa Pan/i, że rodzice dzieci objętych szczepieniem otrzymują wystarczające informacje na temat możliwych skutków ubocznych szczepionek.
a) tak, zdecydowanie się zgadzam
b) raczej się zgadzam
c) nie mam jednoznacznej opinii na ten temat.
d) raczej się nie zgadzam
e) zdecydowanie się nie zgadzam się

44. Czy przed szczepieniem swojego dziecka otrzymywał/a Pan/i zrozumiałe i wystarczające informacje od lekarza na temat szczepienia?
a) tak, zawsze
b) tak, tylko w niektórych przypadkach
c) nie, nigdy
d) nie pamiętam

45. Czy pandemia COVID-19 zmieniła Pana/i pogląd na potrzebę edukacji w zakresie szczepień (nie tylko przeciwko COVID-19)?
a) tak, pandemia zmieniła mój pogląd na potrzebę edukacji szczepień (nie tylko przeciwko COVID-19)
b) tak, częściowo zmieniła mój pogląd na potrzebę edukacji szczepień (nie tylko przeciwko COVID-19)
c) nie mam na ten temat zadania
d) raczej nie, edukacja nie musi być zmieniana
e) nie, edukacja nie musi być zmieniana

46. Czy uważa Pan/Pani, że personel medyczny powinien bardziej angażować się w edukację rodziców na temat szczepień?
a) tak, zdecydowanie
b) nie, obecny poziom edukacji jest wystarczający
c) nie mam zdania

47. Czy czuł/a się Pan/Pani odpowiednio przygotowany/a do podjęcia decyzji o szczepieniu swojego dziecka?
a) tak, czułem/am się dobrze poinformowany/a
b) tak, ale niektóre informacje były niejasne
c) nie, brakowało mi pełnych informacji
d) nie, nie czułem/am się przygotowany/a

VI. Wiedza na temat szczepień

48. Ile chorób obejmuje aktualny obowiązkowy Program Szczepień Ochronnych w Polsce?
a) mniej niż 8
b) około 12 lub 13
c) ponad 20
d) nie wiem

49. Które szczepienie jest wykonywane u dziecka już w pierwszej dobie życia?
a) przeciw odrze
b) przeciw gruźlicy (BCG) i wzw B
c) przeciw meningokokom
d) nie wiem

50. Które z poniższych szczepień jest obowiązkowe w Polsce?
a) przeciw meningokokom
b) przeciw kleszczowemu zapaleniu mózgu
c) przeciw odrze, śwince i różyczce (MMR)
d) nie wiem

51. Szczepienia obowiązkowe w Polsce są:
a) zawsze odpłatne
b) częściowo refundowane
c) bezpłatne
d) nie wiem

52. Do jakiego wieku realizowane są obowiązkowe szczepienia dzieci i młodzieży?
a) do 6. roku życia
b) do 15. roku życia
c) do 19. roku życia
d) nie wiem

53. Które szczepienie należy do szczepień zalecanych, a nie obowiązkowych?
a) przeciw wirusowemu zapaleniu wątroby typu B
b) przeciw meningokokom
c) przeciw poliomyelitis (polio)
d) nie wiem

54. Szczepienie przeciwko ospie wietrznej jest obowiązkowe:
a) dla wszystkich dzieci
b) tylko dla wybranych grup dzieci
c) nie jest dostępne w Polsce
d) nie wiem

55. Szczepienia przeciwko błonicy, tężcowi i krztuścowi wykonywane są:
a) jednorazowo
b) w kilku dawkach zgodnie z kalendarzem
c) tylko w wieku dorosłym
d) nie wiem

56. Szczepienia przeciw pneumokokom w Polsce są:
a) zalecane i płatne
b) obowiązkowe i bezpłatne
c) nie wiem

57. Wybór szczepionki skojarzonej (np. 5w1 lub 6w1) zamiast standardowej:
a) nie jest możliwy
b) jest możliwy i zawsze bezpłatny

c) jest możliwy, ale zazwyczaj odpłatny
d) nie wiem

VII. Proszę o odniesienie się do następujących stwierdzeń (skala VHS)

58. Szczepienia są ważne dla mojego zdrowia
a) Zdecydowanie się nie zgadzam
b) Nie zgadzam się
c) Nie mam zdania
d) Zgadzam się
e) Zdecydowanie się zgadzam

59. Szczepienia są skuteczne.
a) Zdecydowanie się nie zgadzam
b) Nie zgadzam się
c) Nie mam zdania
d) Zgadzam się
e) Zdecydowanie się zgadzam

60. Zaszczepienie się jest ważne dla zdrowia innych osób w moim otoczeniu
a) Zdecydowanie się nie zgadzam
b) Nie zgadzam się
c) Nie mam zdania
d) Zgadzam się
e) Zdecydowanie się zgadzam

61. Wszystkie rutynowe szczepienia zalecane przez lekarzy są korzystne
a) Zdecydowanie się nie zgadzam
b) Nie zgadzam się
c) Nie mam zdania
d) Zgadzam się
e) Zdecydowanie się zgadzam

62. Nowe szczepionki niosą ze sobą więcej zagrożeń niż starsze.
a) Zdecydowanie się nie zgadzam
b) Nie zgadzam się
c) Nie mam zdania
d) Zgadzam się
e) Zdecydowanie się zgadzam

63. Informacje o szczepionkach, które otrzymuję od pracowników ochrony zdrowia są rzetelne i wiarygodne
a) Zdecydowanie się nie zgadzam
b) Nie zgadzam się
c) Nie mam zdania
d) Zgadzam się
e) Zdecydowanie się zgadzam

64. Szczepienia to dla mnie dobry sposób zabezpieczenia się przed chorobami
a) Zdecydowanie się nie zgadzam
b) Nie zgadzam się
c) Nie mam zdania
d) Zgadzam się
e) Zdecydowanie się zgadzam

65. Jeśli chodzi o szczepienia, to zazwyczaj kieruję się zaleceniami lekarza lub innego pracownika ochrony zdrowia
a) Zdecydowanie się nie zgadzam
b) Nie zgadzam się
c) Nie mam zdania
d) Zgadzam się

e) Zdecydowanie się zgadzam

66. Obawiam się poważnych skutków ubocznych szczepionek
a) Zdecydowanie się nie zgadzam
b) Nie zgadzam się
c) Nie mam zdania
d) Zgadzam się
e) Zdecydowanie się zgadzam

67. Nie potrzebuję szczepień przeciwko chorobom, które nie są już częste:

a) Zdecydowanie się nie zgadzam
b) Nie zgadzam się
c) Nie mam zdania
d) Zgadzam się
e) Zdecydowanie się zgadzam

ENGLISH TRANSLATION

Translation of the original Polish-language questionnaire used for data collection

Parental Attitudes toward Childhood and Adolescent Vaccination in Poland after the COVID-19 Pandemic

Dear Sir/Madam,

Thank you for completing the following survey. The aim of the study is to assess the attitudes of parents of children and adolescents toward vaccination after the COVID-19 pandemic. The survey is anonymous and will be used exclusively for research purposes.

I. DEMOGRAPHIC AND SOCIAL INFORMATION

1. Sex:

a) female
b) male

2. Age in years:

………
…

3. Education

a) primary education
b) vocational education
c) secondary education
d) post-secondary education
e) higher education

4. Marital status

a) single
b) in a relationship

5. Place of residence (city/rural area)

a) rural area
b) town/city with up to 50,000 inhabitants
c) town/city with more than 50,000 and up to 100,000 inhabitants
d) city with more than 100,000 inhabitants

6. Place of residence (voivodeship)

a) dolnośląskie
b) kujawsko-pomorskie
c) lubelskie
d) lubuskie
e) łódzkie
f) małopolskie
g) mazowieckie
h) opolskie
i) podkarpackie
j) podlaskie
k) pomorskie
l) śląskie
m) świętokrzyskie
n) warmińsko-mazurskie
o) wielkopolskie
p) zachodniopomorskie

7. Financial situation

a) very good
b) good
c) poor

8. Number of children

a) 1
b) 2
c) 3
d) 4
e) more than 4
f) I do not have children under 18 years of age

9. Sex of the child/children

- child no. 1 - ……………………………………………………………………………………………
- child no. 2 - ……………………………………………………………………………………………
- child no. 3 - ……………………………………………………………………………………………
- child no. 4 - ……………………………………………………………………………………………
- child no. 5 - ……………………………………………………………………………………………

10. Age of the child (in years):

- child no. 1 - ……………………………………………………………………………………………
- child no. 2 - ……………………………………………………………………………………………
- child no. 3 - ……………………………………………………………………………………………
- child no. 4 - ……………………………………………………………………………………………
- child no. 5 - ……………………………………………………………………………………………

II. INFORMATION ON VACCINATION OF THE CHILD/CHILDREN

11. Do you vaccinate your child/children according to the current vaccination schedule?

a) yes
b) no — please proceed to question 13

12. Why did you decide to vaccinate your child? (multiple responses may be selected)

a) I believe that vaccinations are necessary for the child's health
b) because they are mandatory
c) I want to protect other children and people around us
d) I trusted the recommendations of a physician or healthcare professional
e) I want to prevent serious diseases and their complications

f) vaccinations are an established and safe method of prevention
g) I am concerned about infectious diseases and their consequences
h) vaccinations increase population immunity (so-called herd immunity)
i) I wanted to avoid formal difficulties (e.g., in kindergarten or school)
j) I concluded that the benefits of vaccination outweigh the potential risks
k) other
Please proceed to question 14.

13. Why did you decide not to vaccinate your child? (multiple responses may be selected)

a) I am concerned about vaccine-related adverse effects
b) I believe that vaccinations are unnecessary
c) I have doubts about vaccine safety
d) I am concerned about the long-term effects of vaccination
e) I believe that the child has sufficient natural immunity
f) I have had negative experiences related to vaccination (in the child and/or family)
g) my child has medical contraindications to vaccination
h) I received conflicting information about vaccination
i) I do not trust pharmaceutical companies that manufacture vaccines
j) the decision resulted from my own beliefs or worldview
k) I am concerned about too many vaccinations being administered within a short period of time
l) vaccinations are inconsistent with my religious beliefs
m) the decision was made after consultation with other parents and/or under the influence of online sources
n) other

14. Do you choose recommended vaccinations for children that are not mandatory (so-called "recommended vaccinations")?

a) yes — please proceed to question 16
b) no
c) only in selected cases

15. Why do you not choose recommended vaccinations for your child? (multiple responses may be selected)

a) I am concerned about vaccine-related adverse effects
b) I believe that recommended vaccinations are unnecessary
c) I have doubts about vaccine safety
d) the child is healthy and does not need additional vaccinations
e) recommended vaccines are usually too expensive
f) the child receives many mandatory vaccinations and does not need recommended vaccinations
g) I am concerned about the long-term effects of vaccination
h) I believe that the child's natural immunity is sufficient
i) I receive conflicting information about recommended vaccinations
j) there is insufficient information from healthcare professionals on this subject
k) I do not trust vaccine manufacturers
l) other

16. Do you choose vaccines offered by the healthcare system, or do you prefer combination vaccines (e.g., 6-in-1) to avoid multiple injections?

a) I choose combination vaccines (e.g., 6-in-1)
b) I prefer vaccines offered by the healthcare system, even if this means more injections
c) I do not know

III. ATTITUDES TOWARD CHILDHOOD VACCINATION

17. Do you believe that vaccination provides the best protection for children against serious diseases?

a) yes, I strongly agree

b) I rather agree
c) I do not have a clear opinion on this matter
d) I rather disagree
e) I strongly disagree

18. Do you have confidence in vaccinations?

a) I have a high level of confidence
b) I rather have confidence
c) I have no opinion on this matter
d) I rather do not have confidence
e) I do not have confidence

19. Do you believe that vaccines for children are safe?

a) completely safe
b) rather safe
c) risky
d) very risky
e) I have no opinion

20. Do you believe that vaccines are important?

a) very important
b) rather important
c) I have no opinion on this matter
d) not as important as they are claimed to be
e) definitely unimportant

21. Do you believe that vaccines are effective?

a) they are very effective
b) they are effective
c) I have no opinion on this matter
d) they are rather ineffective
e) they are definitely ineffective

22. Do you believe that, thanks to vaccination, many dangerous diseases that were once common have now almost disappeared?

a) yes, I strongly agree
b) I rather agree
c) I do not have a clear opinion on this matter
d) I rather disagree
e) I strongly disagree

23. Do you believe that, overall, vaccinating children provides more benefits than risks?

a) yes, I strongly agree
b) I rather agree
c) I do not have a clear opinion on this matter
d) I rather disagree
e) I strongly disagree

24. Please respond to the following statement: "Vaccinating children is consistent with my religious beliefs":

a) yes, I strongly agree
b) I rather agree
c) I do not have a clear opinion on this matter
d) I rather disagree
e) I strongly disagree

25. Please respond to the following statement: “Vaccination not only protects the person who receives it, but also other people”:

a) yes, I strongly agree
b) I rather agree
c) I do not have a clear opinion on this matter
d) I rather disagree
e) I strongly disagree

26. Please respond to the following statement: “Vaccines for children can lead to serious developmental disorders such as autism.”

a) yes, I strongly agree
b) I rather agree
c) I do not have a clear opinion on this matter
d) I rather disagree
e) I strongly disagree

27. Please respond to the following statement: “Vaccination may weaken a child’s natural immunity.”

a) yes, I strongly agree
b) I rather agree
c) I do not have a clear opinion on this matter
d) I rather disagree
e) I strongly disagree

28. Please respond to the following statement: “I believe it is better to allow a child to go through an infectious disease, such as measles or chickenpox, than to vaccinate, because after a few days of illness the child gains natural immunity for life.”

a) yes, I strongly agree
b) I rather agree
c) I do not have a clear opinion on this matter
d) I rather disagree
e) I strongly disagree

29. Please respond to the following statement: “Vaccines for children can cause serious adverse effects and complications”:

a) yes, I strongly agree
b) I rather agree
c) I do not have a clear opinion on this matter
d) I rather disagree
e) I strongly disagree

30. Please respond to the following statement: “Children receive too many vaccines during the first years of life”:

a) yes, I strongly agree
b) I rather agree
c) I do not have a clear opinion on this matter
d) I rather disagree
e) I strongly disagree

31. Please respond to the following statement: “There are too many vaccinations in the current vaccination schedule—the number of vaccinations should be lower”:

a) yes, I strongly agree
b) I rather agree
c) I do not have a clear opinion on this matter
d) I rather disagree
e) I strongly disagree

32. Please respond to the following statement: "Vaccinations are promoted not because they are genuinely needed, but because doing so benefits pharmaceutical companies":

a) yes, I strongly agree
b) I rather agree
c) I do not have a clear opinion on this matter
d) I rather disagree
e) I strongly disagree

IV. ATTITUDES TOWARD THE PANDEMIC AND ITS IMPACT ON VACCINATION DECISIONS

33. Before the pandemic, were you a supporter of vaccination?

a) yes, I supported all vaccinations
b) rather yes
c) I do not have a clear opinion on this matter
d) rather no
e) no, I was not a supporter of vaccination

34. Are you currently a supporter of vaccination?

a) yes, I am a supporter of vaccination
b) rather yes
c) I do not have a clear opinion
d) rather no
e) no, I am not a supporter of vaccination

35. Did the COVID-19 pandemic influence your attitudes toward vaccination?

a) yes, definitely
b) yes, to some extent
c) I do not have a clear opinion (please proceed to question 37)
d) rather no (please proceed to question 37)
e) no, it had no impact (please proceed to question 37)

36. In what way did the COVID-19 pandemic influence your attitudes toward vaccination?

a) the pandemic reinforced my belief that vaccinations are necessary
b) the pandemic caused me to become a supporter of vaccination
c) the pandemic reinforced my belief that vaccinations are unnecessary
d) the pandemic caused me to become an opponent of vaccination
e) the pandemic had no impact on my attitude toward vaccination

37. Did your assessment of the role of vaccination in combating the COVID-19 pandemic change as a result of experiences related to the pandemic?

a) definitely yes
b) rather yes
c) rather no
d) definitely no
e) I cannot assess / difficult to say

38. Did your trust in the healthcare system increase or decrease as a result of the COVID-19 pandemic?

a) definitely increased
b) rather increased
c) it had no impact
d) rather decreased
e) definitely decreased

39. Did you receive a COVID-19 vaccine?

a) yes, during the pandemic I considered it the right thing to do — please proceed to question 41

b) yes, I did so only because otherwise I would not have been able to perform my professional work — please proceed to question 41
c) no

40. Why did you not get vaccinated against COVID-19? (multiple responses may be selected)

a) concerns about vaccine safety
b) lack of confidence in vaccine effectiveness
c) problems with vaccine availability
d) ideological or religious opposition
e) lack of understanding or knowledge about the vaccination process
f) low trust in public institutions
g) I generally do not trust vaccines
h) other

41. Did experiences related to the pandemic (e.g., health concerns, difficulties related to treating patients) lead you to adopt a more positive attitude toward vaccination?

a) definitely yes
b) rather yes, to some extent
c) I do not know, difficult to say
d) rather no
e) no, it had no impact

V. EDUCATION AND INFORMATION ABOUT VACCINATION

42. Which of the following sources of information have the greatest influence on shaping your attitudes and opinions about vaccination?

a) healthcare professionals (e.g., physicians, nurses)
b) media (television, radio, press)
c) the Internet (websites, discussion forums)
d) academic lectures
e) opinions of friends and family

43. Do you believe that parents of children eligible for vaccination receive sufficient information about possible vaccine-related adverse effects?

a) yes, I strongly agree
b) I rather agree
c) I do not have a clear opinion on this matter
d) I rather disagree
e) I strongly disagree

44. Before your child was vaccinated, did you receive understandable and sufficient information about the vaccination from a physician?

a) yes, always
b) yes, but only in some cases
c) no, never
d) I do not remember

45. Did the COVID-19 pandemic change your view on the need for vaccination education (not only regarding COVID-19)?

a) yes, the pandemic changed my view on the need for vaccination education (not only regarding COVID-19)
b) yes, it partially changed my view on the need for vaccination education (not only regarding COVID-19)
c) I have no opinion on this matter
d) rather no, education does not need to be changed
e) no, education does not need to be changed

46. Do you believe that healthcare professionals should be more involved in educating parents about vaccination?

a) yes, definitely
b) no, the current level of education is sufficient
c) I have no opinion

47. Did you feel adequately prepared to make a decision about vaccinating your child?

a) yes, I felt well informed
b) yes, but some information was unclear
c) no, I lacked complete information
d) no, I did not feel prepared

VI. KNOWLEDGE ABOUT VACCINATION

48. How many diseases are covered by the current mandatory National Immunization Program in Poland?

a) fewer than 8
b) approximately 12 or 13
c) more than 20
d) I do not know

49. Which vaccination is administered to a child as early as the first day of life?

a) against measles
b) against tuberculosis (BCG) and hepatitis B
c) against meningococcal disease
d) I do not know

50. Which of the following vaccinations is mandatory in Poland?

a) against meningococcal disease
b) against tick-borne encephalitis
c) against measles, mumps, and rubella (MMR)
d) I do not know

51. Mandatory vaccinations in Poland are:

a) always paid for by the patient
b) partially reimbursed
c) free of charge
d) I do not know

52. Up to what age are mandatory vaccinations for children and adolescents administered?

a) up to 6 years of age
b) up to 15 years of age
c) up to 19 years of age
d) I do not know

53. Which vaccination is recommended rather than mandatory?

a) against hepatitis B
b) against meningococcal disease
c) against poliomyelitis (polio)
d) I do not know

54. Vaccination against varicella is mandatory:

a) for all children
b) only for selected groups of children
c) it is not available in Poland
d) I do not know

55. Vaccinations against diphtheria, tetanus, and pertussis are administered:

a) once
b) in several doses according to the vaccination schedule
c) only in adulthood
d) I do not know

56. Pneumococcal vaccinations in Poland are:

a) recommended and paid for by the patient
b) mandatory and free of charge
c) I do not know

57. Choosing a combination vaccine (e.g., 5-in-1 or 6-in-1) instead of the standard vaccine:

a) is not possible
b) is possible and always free of charge
c) is possible, but usually requires payment
d) I do not know

VII. PLEASE RESPOND TO THE FOLLOWING STATEMENTS (VHS SCALE)

58. Vaccinations are important for my health

a) Strongly disagree
b) Disagree
c) I have no opinion
d) Agree
e) Strongly agree

59. Vaccinations are effective.

a) Strongly disagree
b) Disagree
c) I have no opinion
d) Agree
e) Strongly agree

60. Being vaccinated is important for the health of other people around me

a) Strongly disagree
b) Disagree
c) I have no opinion
d) Agree
e) Strongly agree

61. All routine vaccinations recommended by physicians are beneficial

a) Strongly disagree
b) Disagree
c) I have no opinion
d) Agree
e) Strongly agree

62. New vaccines carry more risks than older vaccines.

a) Strongly disagree
b) Disagree
c) I have no opinion
d) Agree
e) Strongly agree

63. The information about vaccines that I receive from healthcare professionals is accurate and reliable

a) Strongly disagree
b) Disagree

c) I have no opinion
d) Agree
e) Strongly agree

64. Vaccination is a good way for me to protect myself from diseases

a) Strongly disagree
b) Disagree
c) I have no opinion
d) Agree
e) Strongly agree

65. When it comes to vaccination, I usually follow the recommendations of a physician or another healthcare professional

a) Strongly disagree
b) Disagree
c) I have no opinion
d) Agree
e) Strongly agree

66. I am concerned about serious vaccine-related adverse effects

a) Strongly disagree
b) Disagree
c) I have no opinion
d) Agree
e) Strongly agree

67. I do not need vaccinations against diseases that are no longer common:

a) Strongly disagree
b) Disagree
c) I have no opinion
d) Agree
e) Strongly agree

End of English translation.
